# A Systematic Review of the Long-Term Effects of Using Smartphone- and Tablet-Based Rehabilitation Technology for Balance and Gait Training and Exercise Programs

**DOI:** 10.3390/bioengineering10101142

**Published:** 2023-09-28

**Authors:** Chihyeong Lee, Jooeun Ahn, Beom-Chan Lee

**Affiliations:** 1 Department of Physical Education, Seoul National University, Seoul 08826, Republic of Korea; chi0412@snu.ac.kr; 2 Institute of Sport Science, Seoul National University, Seoul 08826, Republic of Korea; 3 Department of Health and Human Performance, University of Houston, Houston, TX 77204, USA

**Keywords:** smartphone, tablet, balance, gait, telerehabilitation, in-home use, long-term exercise, carry-over effects

## Abstract

Recent advances in wearable motion sensors, mobile devices, the Internet of Things, and telecommunications have created new potential for telerehabilitation. Recognizing that there is no systematic review of smartphone- or tablet-based balance and gait telerehabilitation technology for long-term use (i.e., four weeks or more), this systematic review summarizes the effects of smartphone- or tablet-based rehabilitation technology on balance and gait exercise and training in balance and gait disorders. The review examined studies written in English published from 2013 to 2023 in Web of Science, Pubmed, Scopus, and Google Scholar. Of the 806 studies identified, 14 were selected, and the National Institutes of Health Quality Assessment Tool for Observational Cohort and Cross-sectional Studies was applied to evaluate methodological quality. The systematic review concluded that all 14 studies found balance and gait performance improvement after four weeks or more of balance and gait telerehabilitation. Ten of the 14 studies found that carry-over effects (improved functional movements, muscle strength, motor capacity, cognition, and reduced fear of falling and anxiety levels) were maintained for weeks to months. The results of the systematic review have positive technical and clinical implications for the next-generation design of rehabilitation technology in balance and gait training and exercise programs.

## 1. Introduction

Balance is the ability to maintain a stable and upright position while stationary or moving, and gait is the movement pattern of the limbs during locomotion. Balance and gait control involve complex processes requiring sensory integration and motor control through the central nervous and musculoskeletal systems. Balance and gait impairments can be caused by musculoskeletal injuries, neurological diseases, and age-related decreases in sensory processing, response time, and neuromusculoskeletal function [1].

Balance and gait impairments affect the quality of life, and falling is the primary cause of injury (e.g., fractures, concussions, etc.) and death, regardless of age and pathology [2,3]. One-third of older adults fall at least once annually, and 10% fall multiple times [4]. Numerous systematic reviews and meta-analyses have documented the benefits of clinical and in-home balance and gait rehabilitation for older adults [5,6,7], stroke survivors [8,9,10], individuals with Parkinson’s disease [11,12,13], and patients with multiple sclerosis [14,15,16]. These programs help to preserve intact sensorimotor abilities, restore dysfunctional sensorimotor abilities, and reweight unimpaired sensory signals in cases of sensory loss. The underlying mechanisms are also used in developing new sensorimotor strategies [17].

While there is evidence supporting the importance of frequent but shorter periods of exercise and motor skill training and their continuation [18,19], compliance (adherence) with in-home rehabilitation programs may decline in the absence of real-time therapeutic feedback [20,21,22]. The solution for many individuals is telerehabilitation consisting of virtual reality or video game-based systems equipped with motion sensors (e.g., inertial measurement unit (IMU)) and depth cameras (e.g., Kinect) or instrumented boards (e.g., Wii Balance Board) [23,24,25,26]. Meanwhile, the rapid development of smartphones and tablets, wearable sensor technology, the Internet of Things (IoT), and telecommunications open new possibilities for telerehabilitation (see [27,28,29] for review). For example, smartphone- and tablet-based balance and gait telerehabilitation using inexpensive, portable mobile apps is gradually replacing virtual reality and video game software. The advantages of the new technology include (1) scalable, cost-effective, and time-effective balance and gait training regimens; (2) tailored treatment and therapies; (3) biofeedback or gamification to enhance motivation and compliance; and (4) timely adjustment and feedback by remote therapists.

A few review articles have documented recent efforts to build smartphone-based telerehabilitation technology to improve balance and gait performance, which typically covers design, development, and preliminary assessment in a laboratory setting [27,28,29]. However, these review articles have not examined the effects of long-term use of smartphone-based telerehabilitation, although multiple review articles have suggested balance and gait exercises for at least four weeks to improve performance in various populations (e.g., older adults and individuals with musculoskeletal injuries and neurological disorders) [30,31,32,33,34]. Furthermore, no systematic review has examined sustained improvement, performance retention, and potential carry-over effects of long-term smartphone- or tablet-based balance and gait telerehabilitation.

Recognizing these limitations, this systematic review aimed to examine: (1) the current status of research on the long-term (i.e., four weeks or more) use of smartphone- or tablet-based technologies and the effects on balance and gait telerehabilitation; (2) changes in balance and gait performance over time; and (3) levels of performance retention and carry-over effects. Application guidelines for long-term use of the technology were developed to support engagement and compliance, improve communications with physicians, therapists, caregivers, and family members, and provide tailored exercise programs.

## 2. Materials and Methods

### 2.1. Search Strategy

This systematic review, conducted in accordance with PRSMA 2020 criteria and guidelines, used the Web of Science, PubMed, and SCOPUS databases and the Google Scholar search engine. The search thread included all possible combinations of “smartphone” or “tablet” and “*rehabilitation” or “training” or “exergame” or “exer-game” and “balance” or “gait”. The thread was modified as needed. A manual search of the reference lists used in previous systematic reviews was also conducted. Searches were limited to data published between 2013 and 2023. Table 1 lists the details.

### 2.2. Inclusion and Exclusion Criteria

Studies were included if they: (1) were published in English, peer-reviewed, and full-text accessible; (2) clinically evaluated improvement of balance and/or gait performance; (3) provided telerehabilitation or in-home balance and/or gait exercises; (4) used smartphone- or tablet-based technology with or without additional sensors for telerehabilitation or in-home use; (5) had experimental protocols for more than four weeks based on the recommended period for gait and gait rehabilitation [30,31,32,33,34]; and (6) had participants with balance and/or gait impairments caused by age, diseases, or clinical conditions.

Studies were excluded if they: (1) developed smartphone- or tablet-based balance and/or gait rehabilitation technology only; (2) evaluated the feasibility and usability of smartphone- or tablet-based balance and/or gait rehabilitation technology only; (3) evaluated smartphone- or tablet-based balance or gait rehabilitation technology used in laboratory settings only; (4) had experimental protocols for less than four weeks; (5) excluded balance and/or gait exercises; (6) developed experimental protocols only; (7) were studies discontinued due to unexpected circumstances (e.g., COVID-19); (8) had healthy young adults only; and (9) were systematic reviews.

### 2.3. Study Selection

**Figure 1:**
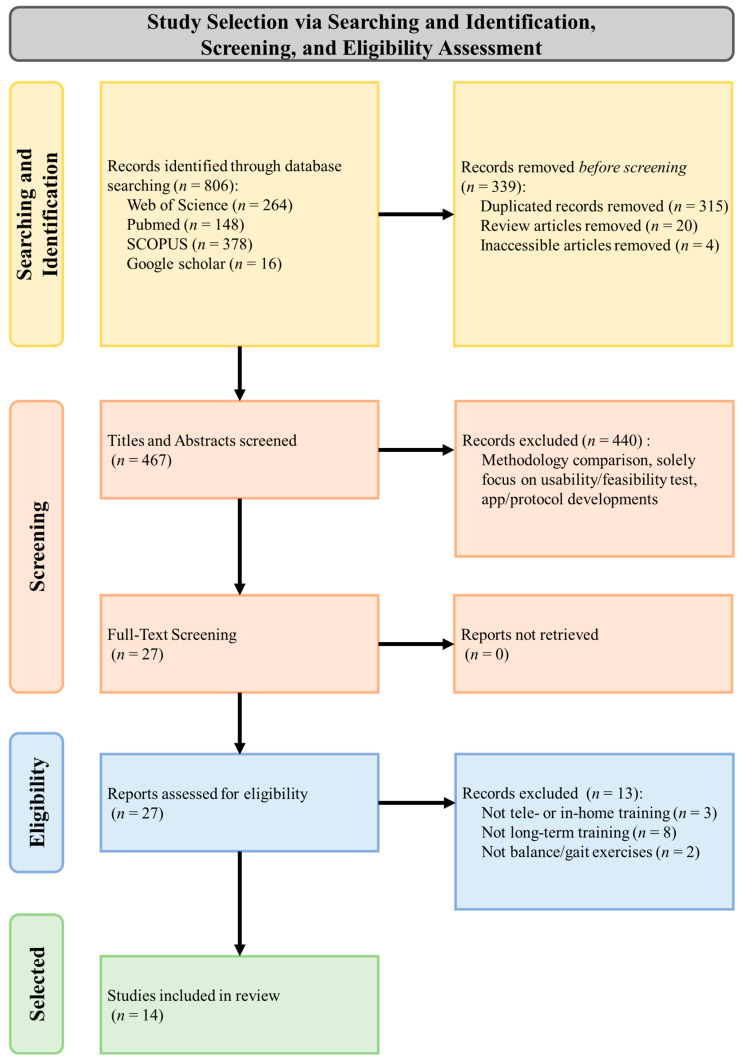
The PRISMA flow diagram presents the progression of the study selection, including how studies were searched, screened, and included in this review.

Figure 1 shows the flow diagram for study selection according to PRISMA guidelines. An initial database search retrieved 806 studies, of which 339 were removed for duplicate records, review articles (including articles not related to smartphone- or tablet-based balance and gait rehabilitation), or inaccessible articles. Of the 467 remaining studies, 440 were removed after title and abstract screening. Of the remaining 27 studies, 13 were removed after full-text screening because they (1) were not telerehabilitation or in-home balance and/or gait training (*n* = 3); (2) were not long-term training (i.e., one-day training in a laboratory setting or a short-term period less than four weeks) (*n* = 8); and (3) did not provide balance or gait exercises (i.e., cardiovascular exercise) (*n* = 2). Therefore, 14 studies were included in this review.

### 2.4. Data Extraction and Tabulation

The information we assessed included: (1) author(s); (2) publication date; (3) participant condition (e.g., diagnosis, disability); (4) sample size; (5) balance and/or gait rehabilitation technology type (smartphone or tablet); (6) additional sensors; (7) exercise and accompanying modality type (biofeedback or gamification); (8) balance and/or gait exercise intervention length; (9) exercise location; and (10) clinical outcome assessment of balance and/or gait rehabilitation.

### 2.5. Methodological Quality

The methodological quality of the studies was assessed with the National Institute of Health’s Quality Assessment Tool for Observational Cohort and Cross-sectional Studies [35]. The tool assesses the responses “Yes”, “No”, and “Other” (“Cannot Determine”, ”Not Reported”, “Not Available”) to 14 questions. The criteria questions from [35] were: (Q1) “Was the research question or objective in this paper clearly stated?”; (Q2) “Was the study population specified and defined?”; (Q3) “Was the participation rate of eligible persons at least 50%?”; (Q4) “Were all the subjects selected or recruited from the same or similar populations (including the same time period) and were inclusion and exclusion criteria for being in the study prespecified and applied uniformly to all participants?”; (Q5) “Was a sample size justification, power description, or variance and effect estimates provided?”; (Q6) “For the analyses in this paper, were the exposure(s) of interest measured prior to the outcome(s) being measured?”; (Q7) “Was the timeframe sufficient so that one could reasonably expect to see an association between exposure and outcome if it existed?”; (Q8) “For exposures that can vary in amount or level, did the study examine different levels of the exposure as related to the outcome (e.g., categories of exposure, or exposure measured as continuous variable)?”; (Q9) “Were the exposure measures (independent variables) clearly defined, valid, reliable, and implemented consistently across all study participants? ”; (Q10) “Was the exposure(s) assessed more than once over time?”; (Q11) “Were the outcome measures (dependent variables) clearly defined, valid, reliable, and implemented consistently across all study participants?”; (Q12) “Were the outcome assessors blinded to the exposure status of participants?”; (Q13) “Was loss to follow-up after baseline 20% or less?”; and (Q14) “Were key potential confounding variables measured and adjusted statistically for their impact on the relationship between exposure(s) and outcome(s)?”

Following the guidelines of the National Institute of Health’s Quality Assessment Tool for Observational Cohort and Cross-sectional Studies, each author independently assessed the methodological quality of all 14 studies. After we assessed the methodological quality and in-depth discussions, the studies were classified into “good”, “fair”, or “low” methodological quality [35]. “Good quality” received “Yes” responses to 8 or more of the 14 questions; “fair quality” received “Yes” responses to 5, 6, or 7 questions; and “low quality” received less than 5 “Yes” responses [36].

## 3. Results

### 3.1. Study Quality

Table 2 reports the results of the methodological quality assessment. The Q12 was not applicable (N/A) and was excluded. All 14 studies received “Yes” responses to 8 or more of the 14 questions, and, therefore, were rated as good overall quality with a low risk of bias. The sample size was the most common shortcoming.

### 3.2. Study Analysis

#### 3.2.1. Participant Cohort

Seven studies had the highest cohort of “older adults”, 4 had Parkinson’s disease, and 3 had hereditary cerebellar ataxia, chronic mild traumatic brain injury (mTBI), and early subacute stroke, respectively. Table 3 lists the details.

#### 3.2.2. Telerehabilitation Technology

Nine studies used smartphone technology for balance or gait rehabilitation, and five used tablet technology. Participants were trained through biofeedback (*n* = 8), recommended exercises (*n* = 4), or exergames (*n* = 2).

Table 4 reports that five studies used visual and vibrotactile biofeedback in real-time as an intervention [39,40,41,45,46]. Two studies used the Smarter Balance System (SBS) [39,40]. They used a Samsung Galaxy A30 (Samsung Electronics Co., Ltd., Suwon, Republic of Korea) and a custom belt consisting of a microcontroller, an inertial measurement unit (IMU), a Bluetooth module, a battery, and four vibration motors for vibrotactile biofeedback. In these two studies [39,40], participants with Parkinson’s disease received dynamic weight-shifting balance exercises (WSBEs) in the anterior-posterior (AP) and medial-lateral (ML) directions. The SBS’s app stored anonymized exercise data in the smartphone’s internal storage and transmitted the stored data to a secure database using Wi-Fi or the Global System for Mobile Communications (GSM).

The other three studies used the same smartphone balance trainer [41,45,46]. The smartphone was equipped with two Apple iPods (sixth-generation iPod touch, 2015) as a sensing unit and a user interface unit, respectively. A custom belt was also designed, which included a “tactor bud” consisting of a controller board, a battery, and four vibration motors (i.e., tactors). The three studies assessed trunk sway by attaching the sensor unit to a participant’s lower back at approximately the L4/L5 spinal level [41,45,46]. The four tactors for vibrotactile biofeedback were aligned with the navel, lumbar spine, and right and left sides of the trunk. Older adults performed customized clinical balance training (CBT) wearing a smartphone balance trainer. When the body tilt angles in the AP and ML directions were greater than a predetermined threshold during exercise, the sensor unit generated audio signals sent to the “tactor bud” to deliver vibrotactile biofeedback. The auditory impulses were processed by the tactor bud, which then actuated the proper tactor to deliver vibrotactile cues. The sensor unit’s app stored anonymized exercise data in the smartphone’s internal storage and transmitted the stored data to a cloud server via Wi-Fi. Additionally, the stored data was sent to a physical therapist, who selected customized (modified) exercises and emailed them weekly to each participant.

Table 4 reports that three studies used auditory biofeedback [43] or visual and auditory multimodal biofeedback [37,44]. One study developed a smartphone app called BeatWalk, including a music-based gait rehabilitation protocol tailored to each participant [43]. During gait training using the Beat-Walk, participants with Parkinson’s disease walked outdoors in a safe area while listening to step-synchronized music. The Beat Walk altered the music’s rhythm to establish spontaneous mutual synchronization with the participant’s gait. Specifically, the music’s tempo was adjusted in real-time to correspond with the participants’ cadence, determined by monitoring the participants’ five most recent footfalls using ankle-worn sensors. The second study used the CuPiD system, consisting of a smartphone (Galaxy S3-mini, Samsung Electronics Co., Ltd., Suwon, Republic of Korea) and IMU sensors [37]. Participants with Parkinson’s disease received gait training. In the first phase of training, they put smartphones in their pockets and received auditory feedback based on the data from the IMU sensors on their shoes. Participants could set training parameters (cadence, stride length, symmetry, gait speed, etc.) in the auditory biofeedback (ABF) app. In the second phase of training, they held smartphones in their hands and received visual feedback based on the data from IMU sensors attached above their ankles. Freezing of gait (FOG)-cue app showed ‘Go’ or ‘No’ signs to participants. The third study used smartphones (Samsung Galaxy S4, Samsung Electronics Co., Ltd., Suwon, Republic of Korea) and headphones [44]. A smartphone was placed on a participant’s lower back at approximately the L4/L5 spinal level during static balance exercises and at approximately the T7/T8 spinal level during dynamic balance exercises. The auditory biofeedback (ABF) application (uABF v1.0, 2016, mHealth Inc., San Diego, CA, USA) measured the smartphone’s linear acceleration to compute body sway and deliver auditory biofeedback about body sway’s acceleration through headphones. Participants received auditory biofeedback when sway exceeded the baseline calibration limitations. Participants with chronic mild traumatic brain injury (mTBI) received balance rehabilitation training with or without using the ABF.

Table 4 reports that four studies used pre-recorded or real-time demonstrations to give interventions to elderly participants [38,42,47,48]. Among them, two studies used pre-recorded demonstrations on tablets [38,42]. ActiveLifestyle is a software app for active aging that aids, monitors, and motivates older individuals during self-directed home-based physical activities [38]. The ActiveLifestyle software included balance and strength exercises, and the participants could select one of three levels: beginner, intermediate, or expert. Participants’ adherence data were computed during the intervention and stored in a central database. The StandingTall app offers individualized and progressive balance and strength exercises to help older adults prevent falls, and it completes goal-setting with participants’ feedback [42]. Participants received an iPad with the StandingTall program included. Balance and cognitive dual-tasking activities were included in the training. The training difficulty increased automatically (in terms of complexity and duration) depending on the participant’s self-reported evaluation of each exercise. Following data transmission to a secure server operated by Neuroscience Research Australia, feedback on participants’ adherence was provided via the app in the form of a weekly counter and a graph, as well as to researchers through a dashboard.

The other two studies used web-based real-time demonstrations as training methods [47,48]. In one study, participants completed supervised resistance and balancing exercises using elastic resistance bands, following the instructor’s instructions on the web browser [47]. Web Real-Time Communication (WebRTC) technology allows the telerehabilitation platform to conduct phone calls, video conversations, and text messages in the browser without requiring any plug-in software. WebRTC is an open-source technology that works with numerous operating systems (e.g., Windows, Android, iOS) and Web browsers (e.g., Chrome, Firefox, Opera, Microsoft Edge). The Biomedical Research Institute of Seoul National University Hospital in Seoul, Korea, collected and stored the exercise data. In the other study, participants gathered in a community center and did flexibility, strength, and balance exercises led by a physical therapist on the screen [48]. The therapist performed exercises in front of a camera at the university lab, which were then projected onto a screen in the community center. Tablets (iPad Air2, Apple, Cupertino, CA, USA) and the web conferencing system called Live On (Japan Media System Corporation, Tokyo, Japan) were used for shooting and receiving exercise videos in real time.

Table 4 reports two studies that used exergames for balance and gait training [49,50]. One study used smartphone-based exergames (Samsung Galaxy J7 Prime 2016, Samsung Electronics Co., Ltd., Suwon, Republic of Korea) controlled by body motions to train participants’ balance ability. A therapist monitored early subacute stroke participants while they performed exergames through a connected web platform, and participants reviewed game scores with a therapist daily at the scheduled session time or afterwards depending on therapist availability. The interaction flow between participants and therapists was designed to sustain connection and increase exercise frequency using the developed platform. Additionally, daily communication was maintained using WhatsApp’s phone app to enhance protocol adherence and respond quickly to any recognized technical problems. The other study used tablet-based exergames to improve balance and cognition in older adults with mild cognitive impairment (MCI) or dementia [50]. They developed Tele-FootX^TM^, an interactive tele-Exergame system that consists of a motion sensor module (LEGSys^TM^, BioSensics, Newton, MA, USA) and a tablet (Galaxy Tab S5e, Samsung Electronics Co., Ltd., Suwon, Republic of Korea) equipped with a custom application. LEGSys^TM^ is equipped with a triaxial accelerometer and gyroscope, a microprocessor, and a Bluetooth module. The user-centered design of the custom application included an intuitive user interface and minimal manual interactions. The custom application delivered balance and cognitive exercises through auditory and visual instructions. Participants confirmed their movements in real time while performing exercises. The exercise progress and adherence were monitored remotely, and the exercise data were stored on the tablet’s internal memory and transferred to a cloud-based server for storage and analysis without the user’s private information.

#### 3.2.3. Training Type, Period, and Location

Table 4 lists the training type, period, and location for all 14 studies. Gait training was used in two studies [37,43], whereas balance training was used in 12 studies [38,39,40,41,42,44,45,46,47,48,49,50]. Among 12 studies using balance training, six studies provided balance training only [39,40,44,45,46,49], and the other six studies provided strength training (*n* = 1) [38], strength and flexibility training (*n* = 1) [48], coordination training (*n* = 1) [41], resistance training (*n* = 1) [47], cognitive training (*n* = 1) [50], or strength and cognitive training (*n* = 1) [42] in addition to balance training. All 14 studies provided four weeks or more of balance and gait training. Although all 14 studies involved at least four weeks of balance and gait training, the training periods were varied. Six weeks of training were the most common (*n* = 6) [37,39,40,41,44,50], followed by 12 weeks (*n* = 2) [38,47], then four weeks (*n* = 2) [43,49], eight weeks (*n* = 2) [45,46], and six months (*n* = 2) [42,48]. Additionally, three studies performed the retention assessments. After completing training, two studies assessed performance retention at one month [37,39], while another study performed retention assessments at one week, one month, and six months [46]. Participants performed balance and gait training at home (*n* = 10) [38,39,40,42,44,45,46,47,49,50], at home and around the home (*n* = 3) [37,41,43], or in a community center (*n* = 1) [48].

#### 3.2.4. Effects of Real-Time Biofeedback

Eight studies included in this review employed real-time biofeedback. Two studies using the SBS system provided visual and vibrotactile multimodal biofeedback for six weeks of balance training [39,40]. The exponential model of cross-correlation (XCORR) and position error (PE) describes balance training performance more effectively than the linear model [39,40]. Exponential trends showed a performance plateau after three weeks and a quasi-steady state performance by the end of six consecutive weeks [40]. After training, limits of stability (LOS) scores increased by 32.1% in the AP direction and 52% in the ML direction, respectively, and the improved LOS were retained for one month [39]. Furthermore, most participants agreed that the benefits of training with the SBS system were to improve their balance and perform exercise regularly (Questions 5 and 6, both scored 4.86 ± 0.38 for 8-point Likert scale questions) [40].

Three studies used the smartphone balance trainer to provide vibrotactile biofeedback for balance training [41,45,46]. One study evaluated the effect of vibrotactile sensory augmentation (SA) on balance performance [45]. After eight weeks of training, the intervention group (IG) had significant improvements in Sensory Organization Test (SOT) scores (14.8%, *p* < 0.01), vestibular reliance (39.6%, *p* < 0.01), Mini Balance Evaluation Systems Test (MiniBESTest)28 (15.8%, *p* < 0.01), MiniBESTest32 (17.2%, *p* < 0.01), Time Up and Go with Cognitive task (TUG-COG) duration (12.7% decrease, *p* < 0.05), and five times Sit to Stands (5×STS) duration (26.1% decrease, *p* < 0.05) [45]. Between groups, IG showed greater improvements compared with the control group (CG) for SOT scores (375%, *p* < 0.05), MiniBESTest28 (1166.7%, *p* < 0.05), and MiniBESTest32 (only IG improved, *p* < 0.05) [45]. Another study observed the retention effects of long-term balance training, which were assessed one week, one month, and six months after training with vibrotactile SA [46]. Both IG and CG significantly improved SOT scores, vestibular reliance, and MiniBESTest scores at the retention assessment. In particular, the improvement in SOT scores improved at one week, one month, and six months (*p* < 0.01, 0.01, and 0.01, respectively), regardless of the group [46]. However, only the IG maintained a Minimal Detectable Change (MDC) of 8 points in the SOT scores at six months [46]. Vestibular reliance increased significantly, with no effect on the group at one week, one month, and six months (*p* < 0.001, 0.001, and 0.001, respectively) [46]. All participants had improved MiniBESTest28 and MiniBESTest32 scores at one week and six months (Mini-BESTest28: *p* < 0.001 and 0.01, respectively; Mini-BESTest32: *p* < 0.01 and 0.03, respectively) [46]. The IG had significantly better scores than the CG at one week and one month for the MiniBest28 (*p* = 0.04 and 0.03, respectively) and MiniBest32 (*p* = 0.01 and 0.05, respectively) scores [46]. In addition, the functional magnetic resonance imaging (fMRI) data demonstrated a shift from pre-training activation in the vestibular cortex to post-training enhanced activity in the brainstem and cerebellum immediately following the balance training with vibrotactile SA [46]. The other study examined the effects of balance training with and without vibrotactile SA (6 weeks of training with and without vibrotactile SA and 12 weeks of crossover design) [41]. After six weeks of training without vibrotactile SA, the Dynamic Gait Index score increased significantly by 14.6% [41]. After six weeks of training with vibrotactile SA, there was a statistically significant decrease in the Scale for the Assessment and Rating of Ataxia (SARA) scores and SARA posture and gait scores of 15.5% and 46.7%, respectively.

One study using the CuPiD system provided visual and auditory multimodal biofeedback for six weeks of gait training [37]. After training, both IG and CG improved significantly on the primary outcomes (comfortable gait and dual-task gait) [37]. For a comfortable gait, gait speed and stride length increased (IG: 9.0%, 6.8%; CG: 5.2%, 4.8%, respectively), and double support time (% of gait cycle time) decreased (IG: 5.6%, CG: 2.5%, respectively) [37]. The improved gait speed, stride length, and double support time were retained for one month [37]. For dual-task gait, gait speed and stride length increased (IG: 13.5%, 8.4%; CG: 5.8%, 5.2%, respectively), and double support time (% of gait cycle time) decreased (IG: 6.3%, CG: 2.5%, respectively) [37]. The improved gait speed and stride length were retained for one month [37]. In addition, the IG significantly improved on balance (MiniBESTest) by 5.5% post-assessment, while the CG showed no significant change [37]. For the 2-Minute Walk Test (2MWT), both IG and CG significantly improved walking distance by 8.2% and 2.5%, respectively [37].

Two studies provided auditory biofeedback [43,44]. One used the BeatWalk-app for four weeks of gait training [43]. The average ratio of the period during which the participants used the BeatWalk-app to the prescribed duration was 78.8%, and 48.7% of all 39 participants (*n* = 19) used the BeatWalk-app for more than 90% of the prescribed duration [43]. After training, the number of falls decreased by 50% during the training program compared to what participants typically experienced in a week (*p* = 0.07) [43]. The number of falls decreased during the last two weeks of the program compared to the first two weeks [43]. In addition, cadence, velocity, and stride length were improved by 3.9% (*p* = 0.01), 2.6% (*p* = 0.01), 3.1% (*p* < 0.01), and 1.6% (*p* = 0.04), respectively [43]. In addition, the Falls Efficacy Scale score decreased by 8.7% (*p* < 0.05) [43]. Furthermore, 35 participants (90.3%) experienced sufficient joy after one week, and 32 maintained the feeling for the last week. Another study provided auditory biofeedback for six weeks of balance training [44]. After training, both the IG and CG groups demonstrated similar medium-effect-sized decreases in Post-Concussion Symptoms (PCSS) (63.3% and 55%, respectively) and large increases in SOT scores (35% and 26.5%, respectively) [44]. The IG showed a trend of larger effect sizes in increasing motor activation (normalized stiffness and damping) and decreasing time delay compared to the CG [44].

#### 3.2.5. Effects of Pre-Recorded or Real-Time Demonstration

Two studies included in this review employed pre-recorded demonstrations [38,42]. One study provided pre-recorded video on a tablet for balance and strength training [38]. After 12 weeks of training, the three groups’ preferred and fast gait speed (i.e., IG, individual IG, and CG) increased by an average of 11.7% and 10.3%, respectively [38]. In addition, adherence between groups was significantly different (IG: 81.9%; individual IG: 71.1%; CG: 48.1%, respectively) [38]. Furthermore, social motivation strategies proved more effective than individual strategies in stimulating the participants to comply with the training plan [38]. Another study used a tablet to provide pre-recorded videos for balance, strength, and cognitive training [42]. After six months of training, adherence was excellent (84.5%), the usability of the app was high (76.7%), and no serious adverse events were reported [42]. In addition, feedback on the app was positive and included suggestions for future updates [42]. Although there was an increase in the primary outcome measure (i.e., gait speed), this increase was not statistically significant [42].

Two studies included in this review employed real-time demonstrations [47,48]. One study used a tablet to provide web-based balance and resistance training [47]. At the end of the training, there were significant increases in the chair stand test for both IG (74.5%, *p* < 0.001) and CG (8.8%, *p* < 0.05) [47]. Berg Balance Scale (BBS) test scores increased significantly for IG (3%, *p* < 0.5) but decreased for CG [47]. In addition, there was a significant interaction effect between group and time on the chair stand test (*p* < 0.001) and BBS (*p* = 0.03) [47]. Furthermore, a significant interaction effect between group and time was found in the scores for fear of falling (*p* = 0.009) [47]. Scores for fear of falling showed a significantly larger decrease among IG participants (7.4%), while those of CG participants increased. Another study used a tablet to provide web-based balance, strength, and flexibility training [48]. At the end of the training, Timed Up and Go test scores differed significantly between the pre-and post-assessments (8.6% decreased, *p* = 0.001). There were also significant differences in BBS scores between the pre-and post-assessments (0.9% increase, *p* < 0.05). Furthermore, 88.9% of participants experienced physical improvement, and 100% experienced increased closeness with others due to the program. Among all participants, 66.7% appreciated making new friends, 66.7% enjoyed exercising daily, and 55.6% felt energized after participating in the telerehabilitation program.

#### 3.2.6. Effects of Gamification

Two studies included in this review employed gamification [49,50]. Among two studies that used exergames for training, one used smartphone-based exergames [49]. As a result of four weeks of balance training using exergames, all participants’ post-assessment scores increased compared to pre-assessment [49]. In particular, BBS, MiniBESTest, and Barthel Index scores for IG increased by 32.4%, 80.7%, and 26.9%, respectively, and those for CG increased by 19.6%, 46.3%, and 6.1%, respectively [49]. However, only the improvement in BBS scores was statistically higher for the IG than the CG [49]. The BBS score variation for IG was 20.20%, while it was only 12.50% for CG [49]. In addition, the average System Usability Scale (SUS) score was 87.5, which can be interpreted as an excellent system usability level (SUS > 80). The other study used a tablet-based exergame system for six weeks of balance and cognitive training [50]. After training, participants reported that tablet-based exergames significantly improved their self-awareness (Q4, 19.4%, *p* = 0.005), overall body functioning (Q7, 15.6%, *p* = 0.015), and flexibility (Q9, 15.6%, *p* = 0.015) [50]. In addition, participants significantly improved their cognition levels and lowered their anxiety levels. In particular, the average Montreal Cognitive Assessment (MoCA) score increased by 9.7%, and the average Beck Anxiety Inventory (BAI) score decreased by 27.6% [50].

## 4. Discussion

This is the first systematic review of studies on the beneficial effects of long-term (i.e., four weeks or longer) balance and gait telerehabilitation using smartphone- or tablet-based technologies. This review suggests that using smartphone- or tablet-based telerehabilitation technology for balance and gait rehabilitation training is effective in improving balance and gait performance, retaining those improvements, and causing carry-over benefits in balance and gait disorders (e.g., older adults, stroke survivors, individuals with Parkinson’s disease, etc.). The subsections below discuss the key benefits identified in all 14 studies.

### 4.1. Increased Accessibility and Convenience

The target populations in all 14 studies were under care at home and performed long-term (4 weeks or longer) balance and gait training in their domestic settings (i.e., in the home or home and around home). Smartphone-based and tablet-based telerehabilitation technology particularly benefitted individuals with mobility limitations and individuals living far from physical therapy facilities and clinics. The target populations used remote telerehabilitation technology to schedule their training, which allowed them to schedule sessions at their preferred time and exempted the healthcare facilities from scheduling the appointment. Potentially, telerehabilitation technology could reduce the cost of healthcare as well as the burden on healthcare facilities [51,52].

### 4.2. Data Tracking and Analysis

Nine of the 14 studies leveraged the features of smartphones and tablets, such as built-in memory and telecommunications [38,39,40,41,42,45,47,49,50]. These studies, in particular, stored exercise performance, progress, and/or adherence data in the smartphone’s or tablet’s built-in memory and transferred them to a secured server. Indeed, data tracking and analysis play crucial roles in smartphone- or tablet-based balance and gait telerehabilitation [53]. Through continuous data tracking, physical therapists or health professionals could monitor participants’ exercise performance and progress objectively and make real-time adjustments to their treatment plans even if they are physically distant from participants. This personalized approach increases the likelihood of achieving positive outcomes after the completion of telerehabilitation exercises. By analyzing data, physical therapists or health professionals could identify effective interventions and make evidence-based decisions, ensuring the rehabilitation program is tailored to each participant’s needs and conditions. In addition, analyzing data over time can reveal patterns and trends that may not be apparent during isolated training sessions at home. It enables physical therapists or health professionals to identify potential challenges where patients struggle while performing telerehabilitation exercises, enabling targeted interventions to address these challenges effectively. Potentially, aggregated anonymized data collection and analysis could be used for research, clinical purposes, and training future physical therapists and health professionals.

### 4.3. Enhanced Engagement and Motivation

Eight, four, and two studies included in this review, respectively, employed real-time biofeedback, video demonstrations, and gamification that could make telerehabilitation exercises more enjoyable, increasing participants’ motivation and adherence to treatment plans and protocols. The modalities for real-time biofeedback used in the eight studies were visual and vibrotactile [39,40,41,45,46], visual and auditory [37], and auditory [43,44], which were provided depending on the participant’s movements measured by either the smartphone’s built-in sensors (e.g., accelerometer, gyroscope, and IMU) or an external motion sensor (i.e., IMU). There is ample evidence that biofeedback-assisted balance exercises are more effective in improving postural stability and gait control than non-biofeedback training [54,55,56,57]. In the eight studies, real-time biofeedback allowed participants to observe and adjust their movements in response to immediate sensory cues, optimizing their movements and muscle activation patterns. Thus, it is reasonable to interpret that real-time biofeedback can promote enhanced motor learning and help participants make necessary corrections during exercises, leading to more effective and efficient telerehabilitation exercises.

Four studies used pre-recorded [38,42] or real-time [47,48] video demonstrations. Pre-recorded video demonstrations allowed repeated viewing, leading the participants to perform the training and exercise correctly and optimize performance. Real-time video demonstrations also allowed physical therapists and health professionals to observe and correct movements. By observing the correct execution of movements, participants could mimic the actions accurately, optimizing the effectiveness of their rehabilitation program.

Two studies used gamification [49,50]. Gamification in telerehabilitation refers to incorporating game-like features and mechanics into rehabilitation exercises and activities, often known as an exergame [58]. Gamification could make rehabilitation exercises more enjoyable and interactive by introducing challenges, rewards, and progress monitoring. In addition, gamification incorporated real-time feedback on participants’ performance, allowing them to make immediate adjustments to improve their performance and track their progress. Notably, gamified telerehabilitation platforms could offer customizable challenges and exercises tailored to each participant’s specific needs and abilities, leading to improved treatment outcomes.

The findings of the 14 studies may support the fact that real-time biofeedback, pre-recorded or video demonstrations, and gamification are engaging and interactive, capturing users’ attention more effectively than traditional text-based instructions alone. These instructions also motivate users to participate actively in rehabilitation exercises and adhere to treatment regimens. Telerehabilitation with real-time biofeedback, video demonstrations, or gamification could potentially improve treatment outcomes and enhance the user experience with the telerehabilitation process.

### 4.4. Improved Outcomes

The 14 studies found that balance and gait performance improvements were maintained for weeks or months. One even found that balance improvements were maintained for up to six months after training, as confirmed by the results of fMRI [46]. Ten studies also reported carry-over effects after people with balance and gait impairments completed lengthy telerehabilitation training exercises, i.e., improved functional movement [37,39,41,45,46,47], motor strength and capacity [37,41,42,48], reduced fear of falling [37,39,43,47], and cognitive function and anxiety level [50]. Long-term and sustained balance and gait rehabilitation may strengthen an individual’s confidence and capacity to perform daily activities, leading to increased involvement in functional tasks and a willingness to take on more challenging activities. Indeed, carry-over effects are crucial as they demonstrate the broader impact of rehabilitation beyond the targeted training tasks, potentially contributing to increased independence and improved quality of life for older adults, stroke survivors, individuals with Parkinson’s disease, etc.

### 4.5. Limitations, Future Work, and Implications of Included Studies

Studies included in this systematic review have additional limitations. First, all 14 studies used fewer than 80 participants (a relatively small sample size) [37,38,39,40,41,42,43,44,45,46,47,48,49,50]; hence, future research with a larger sample size needs to be conducted. Second, there is an inhomogeneity of participants across all 14 studies (e.g., older adults, individuals with neurological disorders, etc.), implying the need to investigate children, vulnerable populations (e.g., non-English speakers, those living in rural areas with limited or no wireless or cellular access), caregivers, including family members, and health care providers. Additionally, participants with more severe symptoms need to be considered [41,44], both the intervention and control groups need to have an equal gender distribution [44], and randomized and blinded trials need to be conducted [37,38]. Third, telerehabilitation protocols need to be improved. For example, future telerehabilitation protocols need to extend the training period (e.g., longer than six weeks) [50], assess carry-over effects after a longer period (e.g., a year or more) [40,47], and include measurements of various balance and gait performance (e.g., instant, static, dynamic, etc.) [44]. Fourth, telerehabilitation technologies need to be improved, considering a lack of remote monitoring [45], limited video quality (i.e., low resolution) [48], and unstable sensor connection and calibration [49]. In addition, all 14 studies used a custom app for smartphones or tablets, implying the need for future research on the effects of free and commercial applications on long-term balance and gait telerehabilitation. Last, the telerehabilitation systems and platforms used in all 14 studies were not approved for sale or did not comply with healthcare regulations, implying the need for future commercialization and regulatory compliance research.

Despite the limitations, the 14 studies included in this systematic review suggested three therapeutically important findings:

1. Rehabilitation exercises that challenge balance and gait, activate neural pathways, and promote neuroplasticity are more likely to generalize the learned skills to similar functional activities.
2. Sensory-augmented or gamified rehabilitation combining balance or gait exercises with cognitive or motor tasks improves the ability to perform activities that require multitasking.
3. Balance and gait rehabilitation exercises with sensory integration challenge the brain to process information from vision, vestibular, proprioception, and other systems, enhancing stability and navigation, particularly in uncertain environments.

When sustained balance and gait rehabilitation strengthens confidence in the ability to perform daily activities, it may lead to more involvement in functional tasks and a willingness to re-engage in previously avoided activities. The systematic review concluded that the 14 studies demonstrated the contributions of long-term balance and gait rehabilitation technology beyond the targeted training tasks for the targeted population.

## 5. Conclusions

This systematic review covered 14 recent studies on the long-term use of smartphone- and tablet-based telerehabilitation technology for balance and gait training and exercise programs performed remotely by people with balance and gait disorders (e.g., older adults, stroke survivors, individuals with Parkinson’s disease, etc.). Telerehabilitation technology embedded with real-time multimodal biofeedback, pre-recorded video demonstrations, and gamification appeared most advantageous for motor learning, treatment outcomes, and carry-over effects. The findings have implications for the development of next-generation technology and both clinical and remote assessments for balance and gait impairments. Indeed, smartphone- and tablet-based telerehabilitation can revolutionize healthcare practice by enhancing accessibility, mobility, engagement, and cost efficiency. However, it also presents challenges related to privacy, equity, and regulatory compliance that must be carefully addressed. Future research will play a crucial role in refining telerehabilitation protocols, assessing their effectiveness, and improving patient outcomes. Future research on smartphone- and tablet-based telerehabilitation may have to prioritize protocol improvements, long-term patient outcomes, addressing access disparities, enhancing services to comply with healthcare regulations, and investigating the ethical and social implications of remote healthcare delivery.

## Figures and Tables

**Table 1:** Searches and search terms.

Web of Science	Title: (“Smartphone” OR “Tablet”) AND (“*rehabilitation” OR “Training” OR “exergame” OR “exer-game”) AND (“balance” OR “gait”) Abstract: (“Smartphone” OR “Tablet”) AND (“rehabilitation” OR “Training” OR “exergame” OR “exer-game”) AND (“balance” OR “gait”) Topic: (“Smartphone” OR “Tablet”) AND (“*rehabilitation” OR “Training” OR “exergame” OR “exer-game”) AND (“balance” OR “gait”)
Pubmed	Title/Abstract: (“Smartphone” OR “Tablet”) AND (“*rehabilitation” OR “Training” OR “exergame” OR “exer-game”) AND (“balance” OR “gait”)
Scopus	Article title, Abstract, Keywords: (“Smartphone” OR “Tablet”) AND (“*rehabilitation” OR “Training”) AND (“balance” OR “gait”)
Google Scholar	With all of the words: (“Smartphone” OR “Tablet”) AND (“rehabilitation” OR “Training” OR “exergame” OR “exer-game”) AND (“balance” OR “gait”)

**Table 2:** Results of the methodological quality assessment.

	Q1	Q2	Q3	Q4	Q5	Q6	Q7	Q8	Q9	Q10	Q11	Q12	Q13	Q14	Overall Quality
Ginis et al. (2016) [37]	Yes	No	Yes	Yes	No	Yes	Yes	Yes	Yes	Yes	Yes	N/A	Yes	Yes	Good
Silveira et al. (2013) [38]	Yes	Yes	Yes	Yes	No	Yes	Yes	Yes	Yes	Yes	Yes	N/A	No	Yes	Good
An et al. (2020) [39]	Yes	Yes	Yes	Yes	No	Yes	Yes	Yes	Yes	Yes	Yes	N/A	NA	No	Good
Lee et al. (2023) [40]	Yes	Yes	No	Yes	No	Yes	Yes	Yes	Yes	Yes	Yes	N/A	No	Yes	Good
Jabri et al. (2022) [41]	Yes	Yes	Yes	Yes	No	Yes	Yes	Yes	Yes	Yes	Yes	N/A	No	Yes	Good
Callisay et al. (2021) [42]	Yes	Yes	Yes	Yes	No	Yes	Yes	Yes	Yes	Yes	Yes	N/A	Yes	Yes	Good
Cochen et al. (2021) [43]	Yes	Yes	Yes	Yes	No	Yes	Yes	Yes	Yes	Yes	Yes	N/A	Yes	Yes	Good
Campbell et al. (2022) [44]	Yes	Yes	Yes	Yes	No	Yes	Yes	Yes	Yes	Yes	Yes	N/A	No	Yes	Good
Bao et al. (2018) [45]	Yes	Yes	Yes	Yes	No	Yes	Yes	Yes	Yes	Yes	Yes	N/A	Yes	Yes	Good
Bao et al. (2022) [46]	Yes	Yes	Yes	Yes	No	Yes	Yes	Yes	Yes	Yes	Yes	N/A	No	Yes	Good
Hong et al. (2018) [47]	Yes	Yes	Yes	Yes	Yes	Yes	Yes	Yes	Yes	Yes	Yes	N/A	No	Yes	Good
Wakasa et al. (2020) [48]	Yes	Yes	Yes	Yes	No	Yes	Yes	Yes	Yes	Yes	Yes	N/A	Yes	Yes	Good
Burgos et al. (2020) [49]	Yes	Yes	Yes	Yes	No	Yes	Yes	Yes	Yes	Yes	Yes	N/A	No	Yes	Good
Park et al. (2022) [50]	Yes	Yes	Yes	Yes	No	Yes	Yes	Yes	Yes	Yes	Yes	N/A	Yes	Yes	Good

Note: Questions from the National Institute of Health’s Quality Assessment Tool for Observational Cohort and Cross-sectional Studies [35].

**Table 3:** Participant cohorts.

Study	Participant Cohort	Age (Years)	Sample Size (Groups)
Ginis et al. (2016) [37]	Parkinson’s disease	Not specified	38 (IG = 20; CG = 18)
Silveira et al. (2013) [38]	Older adults	75 ± 6	33 (Individual IG = 11; Social IG = 12; CG = 10)
An et al. (2020) [39]	Parkinson’s disease	75.5 ± 4.9	2 (N/A)
Lee et al. (2023) [40]	Parkinson’s disease	50–75	7 (N/A)
Jabri et al. (2022) [41]	Hereditary cerebellar ataxia	47 ± 12	7 (N/A)
Callisay et al. (2021) [42]	Older adults	72.8 ± 7	77 (N/A)
Cochen et al. (2021) [43]	Parkinson’s disease	65 ± 9	39 (N/A)
Campbell et al. (2022) [44]	Chronic mild traumatic brain injury (mTBI)	40.9 ± 11	31 (IG = 16; CG = 15)
Bao et al. (2018) [45]	Older adults	75.6 ± 4.9	12 (IG = 6; CG = 6)
Bao et al. (2022) [46]	Older adults	75.4 ± 4.7	15 (IG = 8; CG = 7)
Hong et al. (2018) [47]	Older adults (with Fall Risk Assessment scores > 14)	68–91	23 (IG = 10; CG = 13)
Wakasa et al. (2020) [48]	Older adults	76.3 ± 3.3	9 (N/A)
Burgos et al. (2020) [49]	Early subacute stroke	46–79	10 (IG = 6; CG = 4)
Park et al. (2022) [50]	Older adults (with mild cognitive impairment or dementia)	68.1 ± 5.4	14 (N/A)

Notes: IG: Intervention group; CG: Control group; N/A: Not Applicable.

**Table 4:** Details technologies, delivery modality, trainings, and outcome measures.

Study	Technology and Additional Sensors	Training Type	Delivery Modality	Training and Assessment Periods	Training Location	Outcome Measures
Ginis et al. (2016) [37]	Smartphone IMU sensors	Gait	Real-time visual and auditory biofeedback	Six weeks Three times/week Pre-, Post-, and Retention 1 month	In and around the home	(1) Gait parameters (gait speed, stride length, DS time) (2) Balance (MiniBESTest, FSST, FES-I) (3) Endurance and physical capacity (2MWT, PASE) (4) FOG severity (NFOG-Q, Ziegler protocol) (5) Disease severity (UPDRS III) (6) Cognitive assessments (CTT A and B, VF scores in sitting and walking) (7) Quality of life (SF-36)
Silveira et al. (2013) [38]	Tablet No additional sensors	Balance, Strength	Pre-recorded demonstration	Twelve weeks Five times/week; Balance training Two times/week; Strength training Pre-, Post-	In-home	(1) Adherence and attrition (2) Preferred and fast gait speed (3) Motivation instruments (self-reported 7-point Likert scale questionnaire) (4) Change of behavior (self-reported TTM questionnaire)
An et al. (2020) [39]	Smartphone IMU sensors and four vibration units	Balance	Real-time visual and vibrotactile biofeedback	Six weeks Three times/week Pre-, Post-, and Retention 1 month	In-home	(1) Exercise performance (XCORR, PE) (2) Regression analysis using linear and exponential models were conducted to estimate average XCORR and PE trends (3) Balance and gait performance and level of fear of falling (LOS, SOT, FES, ABC, DGI)
Lee et al. (2023) [40]	Smartphone IMU sensors and four vibration units	Balance	Real-time visual and vibrotactile biofeedback	Six weeks Three times/week Pre-, Post-	In-home	(1) Exercise performance (XCORR, PE) (2) Regression analysis using linear and exponential models were conducted to estimate the trends of the average XCORR and PE (3) TAM questionnaire (8-point Likert scale questionnaire averaged across the participants)
Jabri et al. (2022) [41]	Smartphone Built-in gyroscope and four vibration units	Balance, Coordination	Real-time vibrotactile biofeedback	Six weeks with biofeedback Five times/week Six weeks without biofeedback Five times/week Pre-, Per- (6 weeks), Post-	In and around-home	Balance and coordination assessments (SARA scores, SARA posture & gait subscores, mCTSIB, DGI, TUG, TUG-m, 5×STS, lower-body strength)
Callisay et al. (2021) [42]	Tablet No additional sensors	Balance, Strength, Cognition	Pre-recorded demonstration	Six months Two h/week Pre-, Post-	In-home	(1) Gait parameters (gait speed, dual-task gait speed) (2) Balance (15s step test, FISCIT-4) (3) Muscle strength (5×STS) (4) Cognition (executive function, memory, attention), mood, and balance confidence (5) Adherence, safety, usability, and feedback
Cochen et al. (2021) [43]	Smartphone IMU sensors	Gait	Real-time auditory biofeedback	Four weeks Five times/week Pre-, Post-	In and around the home	(1) Disease severity (Hoehn and Yahr scale) (2) Fall risk (FES) (3) Balance (MiniBESTest) (4) Global cognitive function (MoCA), depressive symptoms (BDI), anxiety (Parkinson’s anxiety scale), apathy (Lille apathy rating scale), fatigue (fatigue severity scale), and quality of life (EQ-5D) (5) Safety and tolerance (daily survey on the number of falls, fatigue, and pain) (6) Observance, usability, and enjoyment (7) Physical activity evaluation (CHAMPS) (8) Gait parameters (6MWT)
Campbell et al. (2022) [44]	Smartphone Built-in acceleration sensor and headphone	Balance	Real-time auditory biofeedback	Six weeks 45 min biweekly Pre-, Post-	In-home	(1) PCSS (2) Balance (SOT, central sensorimotor integration test)
Bao et al. (2018) [45]	Smartphone Built-in gyroscope and four vibration units	Balance	Real-time vibrotactile biofeedback	Eight weeks Three times/week Pre-, Per (4 weeks), Post-	In-home	Balance performance (ABC, SOT, MiniBEST, 5×STS, FSST, FRT, Gait Speed Test, TUG, TUG-COG)
Bao et al. (2022) [46]	Smartphone Built-in gyroscope and four vibration units	Balance	Real-time vibrotactile biofeedback	Eight weeks Three times/week Pre-, Retention 1 (1 week), Retention 2 (1 month), Retention 3 (6 months) fMRI assessment Pre-, Retention (1 week)	In-home	Balance performance (ABC, SOT, MiniBEST, 5×STS, FSST, FRT, Gait Speed Test, TUG, TUG-COG, MDC)
Hong et al. (2018) [47]	Tablet No additional sensors	Balance, Resistance	Real-time demonstration	Twelve weeks (3 times/week) Pre-, Post-	In-home	(1) Body composition (2) Physical function parameters (SFT, BBS) (3) Psychological factors (Korean Falls Efficacy Scale scores, Fear of Falling Questionnaire scores)
Wakasa et al. (2020) [48]	Tablet No additional sensors	Balance, Strength, Flexibility	Real-time demonstration	Six months (60–70 min biweekly) Pre-, Post-	Community center	(1) Motor function–balance and gait (LES, TUG, BBS) (2) Maximal isotonic strengthening of the knee extensors (3) Health status (SF-36) (4) Questionnaire on motivation and perceptions of benefits from participation
Burgos et al. (2020) [49]	Smartphone No additional sensors	Balance	Gamification	Four weeks Nine times/week Pre-, Post-	In-home	(1) Balance performance (BBS, MiniBESTest) (2) Functional independence (BI) (3) System Usability Scale
Park et al. (2022) [50]	Tablet No additional sensors	Balance, Cognition	Gamification	Six weeks Two times/week Pre-, Post-	In-home	(1) Acceptance (TAM questionnaire) (2) Cognition and anxiety level (MoCA, BAI)

Notes: 2MWT: 2-Minute Walk Test; 6MWT: 6-Minute Walk Test; 5×STS: Five-times Sit-To-Stand Test; ABC: Activities-specific Balance Confidence; BAI: Beck Anxiety Inventory; BDI: Beck Depression Inventory; BBS: Berg Balance Scale test; BI: Barthel Index; CHAMPS: questionnaire developed for Community Healthy Activities Model Program for Seniors; CTT: Color Trail Test; DGI: Dynamic Gait Index; DS: Double Stance; FES: Falls Efficacy Scale; FES-I: Falls Efficacy Scale-International; FISCIT-4: Frailty and Injuries Cooperative Studies of Intervention Techniques test; FRT: Functional Reach Test; FSST: Four-Square Step Test; IMU: Inertial measurement unit; LES: Lower Extremity Strength; LOS: Limit Of Stability; mCTSIB: modified Clinical Test of Sensory Interaction in Balance; MDC: Minimal Detectable Change; MDS-UPDRS: Movement Disorder Society-Unified Parkinson’s Disease Rating Scale; MiniBESTest: Mini-Balance Evaluation Systems Test; MoCA: Montreal Cognitive Assessment; NFOG-Q: New Fear Of Gait Questionnaire; PASE: Physical Activity Scale for the Elderly; PCSS: Post-Concussion Symptom; PE: Position Error; SARA: Scale for Assessment and Rating of Ataxia; SF-36: Short Form 36 health survey; SFT: Senior Fitness Test; SOT: Sensory Organization Test; SUS: System Usability Scale; TAM: Technology Acceptance Model; TTM: Transtheoretical Model; TUG: Timed Up and Go; TUG-m: Timed Up and Go with Motor Task; TUG-COG: Timed Up and Go with Cognitive Task; UPDRS: Unified Parkinson’s Disease Rating Scale; VF: Verbal Fluency; XCORR: Cross-Correlation.

## Data Availability

Data are available in the manuscript.
